# Atomistic-Based Fatigue Property Normalization Through Maximum A Posteriori Optimization in Additive Manufacturing

**DOI:** 10.3390/ma18143332

**Published:** 2025-07-15

**Authors:** Mustafa Awd, Lobna Saeed, Frank Walther

**Affiliations:** 1 Institute for Informatics and Automation (IIA), Bremen City University of Applied Sciences (HSB), Flughafenallee 10, 28199 Bremen, Germany; 2 Testia GmbH, Airbus Group, Cornelius-Edzard-Straße 15, 28199 Bremen, Germany; 3 Crystallography & Geomaterials Research Group, Faculty of Geosciences, University of Bremen, Klagenfurter Straße 2-4, 28359 Bremen, Germany; lobna@uni-bremen.de; 4 Chair of Materials Test Engineering (WPT), Faculty of Mechanical Engineering, TU Dortmund University, Baroper Str. 303, 44227 Dortmund, Germany; frank.walther@tu-dortmund.de

**Keywords:** additive manufacturing, fatigue prediction, atomistic modeling, microstructural heterogeneity, Bayesian optimization

## Abstract

This work presents a multiscale, microstructure-aware framework for predicting fatigue strength distributions in additively manufactured (AM) alloys—specifically, laser powder bed fusion (L-PBF) AlSi10Mg and Ti-6Al-4V—by integrating density functional theory (DFT), instrumented indentation, and Bayesian inference. The methodology leverages principles common to all 3D printing (additive manufacturing) processes: layer-wise material deposition, process-induced defect formation (such as porosity and residual stress), and microstructural tailoring through parameter control, which collectively differentiate AM from conventional manufacturing. By linking DFT-derived cohesive energies with indentation-based modulus measurements and a MAP-based statistical model, we quantify the effect of additive-manufactured microstructural heterogeneity on fatigue performance. Quantitative validation demonstrates that the predicted fatigue strength distributions agree with experimental high-cycle and very-high-cycle fatigue (HCF/VHCF) data, with posterior modes and 95 % credible intervals of ${\hat{\sigma }}_{f}^{\mathrm{AlSi}10\mathrm{Mg}}=86_{-7}^{+8}\;\mathrm{MPa}$ and ${\hat{\sigma }}_{f}^{\mathrm{Ti}–6\mathrm{Al}–4V}=115_{-9}^{+10}\;\mathrm{MPa}$, respectively. The resulting Woehler (S–N) curves and Paris crack-growth parameters envelop more than 92 % of the measured coupon data, confirming both accuracy and robustness. Furthermore, global sensitivity analysis reveals that volumetric porosity and residual stress account for over 70 % of the fatigue strength variance, highlighting the central role of process–structure relationships unique to AM. The presented framework thus provides a predictive, physically interpretable, and data-efficient pathway for microstructure-informed fatigue design in additively manufactured metals, and is readily extensible to other AM alloys and process variants.

## 1. Introduction

Fatigue remains the dominant root cause of service failures in metallic components that experience cyclic loading, accounting for more than 80% of in-service damage across the energy, transportation and biomedical sectors [1]. The widespread adoption of additive manufacturing (AM) exacerbates this challenge because the layer-wise process inherently seeds a hierarchy of flaws (residual porosity, lack-of-fusion voids, anisotropic grains and process-frozen residual stresses) that accelerate crack initiation and diminish endurance limits relative to wrought counterparts [2,3]. Although post-process treatments such as hot-isostatic pressing or surface polishing can partially restore fatigue performance [4], the scatter of life data within a single build still spans at least one order of magnitude, frustrating certification efforts and hindering broader industrial uptake [5]. Consequently, there is an urgent need for transferrable, uncertainty-aware predictive frameworks that transform high-fidelity physics and limited test data into reliable estimates of fatigue life.

**Limitations of current fatigue-life models.** Classical design methodologies rely on empirical *S*–*N* or ε–*N* curves derived from large coupon campaigns. These methodologies fail when transferred to different AM machines, powders or parameter sets because they neglect the mechanistic link between process-induced defect populations and crack-tip driving forces [6,7]. Multiscale crystal-plasticity and phase-field simulations have begun to bridge this gap by resolving grain morphology and slip localisation [8]; yet they remain extremely data-hungry, requiring dozens of poorly constrained temperature- and rate-dependent material parameters. Data-driven surrogates based on extreme-gradient boosting or physics-guided neural networks improve computational efficiency and can interpolate within known parameter envelopes [9,10]. Nevertheless, their accuracy deteriorates under covariate shift when the scan vector, alloy chemistry, or post-processing deviates from the training domain [11].

**Density functional theory as an atomistic anchor.** Density Functional Theory (DFT) offers an ab initio route to quantify surface energy $\gamma_{s}$, cohesive energy $U_{0}$, stacking-fault energy and Peierls barrier with predictive accuracy [12,13]. These atomistic energetics govern crack nucleation, cleavage resistance and dislocation-mediated shielding; yet they are rarely injected into continuum fracture and fatigue models. Recent work has demonstrated that DFT-derived $\gamma_{s}$ and $U_{0}$ furnish a lower-physics-bound for the critical strain-energy release rate $G_{c}$, which in turn sets the Griffith toughness $K_{\mathrm{Ic}}$ of brittle solids [14,15]. By systematically computing these quantities for alloy design spaces—Ti-6Al-4V, AlSi10Mg, In718 and emerging high-entropy alloys—the present study creates an atomistically informed catalogue of fracture resistance that is agnostic to processing history.

**From atomistically derived toughness to fatigue crack growth.** The fracture mechanics community has long recognised the link between $K_{\mathrm{Ic}}$ and the threshold stress-intensity range $\Delta K_{\mathrm{th}}$ that demarcates non-propagating cracks; Tanaka and Kuroda formulated empirical scaling relationships where $\Delta K_{\mathrm{th}}\;\propto \;K_{\mathrm{Ic}}{\left(1-\rho \right)}^{m}$, with ρ a plastic-strain ratio at the crack tip. Embedding DFT-calculated $K_{\mathrm{Ic}}$ into such relations circumvents the need for extensive threshold measurements and provides a transferable entry point for Paris-law calibration [16,17]. Furthermore, the Rice–Thomson criterion ties the propensity for a dislocation-shielded crack tip to the ratio of surface energy to unstable stacking-fault energy—both accessible from DFT—thereby rationalising observed variations in Paris exponent *m* among different alloys [18].

**Bayesian calibration and the MAP bottleneck.** Even with physics-based priors, fatigue-life predictions inherit appreciable uncertainties from measurement noise, process variability and model form. Bayesian inference provides a principled framework to propagate these uncertainties; however, the marginalisation integral in Bayes’ rule is analytically intractable for high-dimensional models. Variational Bayes, Laplace approximations, and Markov chain Monte Carlo (MCMC) have been employed to approximate the posterior but often at prohibitive computational cost for design loops [19,20]. Maximum A Posteriori (MAP) estimation offers a pragmatic compromise, delivering the modal parameter set while retaining prior regularisation [21]. Recent extensions such as the State-Augmentation for Marginal Estimation (SAME) strategy accelerate MAP evaluation in latent-variable models [20], and log-concave-prior guarantees affirm that MAP solutions remain proper Bayes estimators under linear inverse problems [18]. Yet, MAP has seen limited deployment in fatigue modelling. This work closes that gap by fusing DFT-based priors with sparse finite element (FE) based crack-growth data through MAP optimisation, thereby producing probability-aware Paris and Woehler curves suitable for light-weight design.

**Complementarity with modern machine learning.** While deep learning has revolutionised process monitoring, defect segmentation [22] and surrogate modelling in AM [23], purely data-driven predictors risk over-fitting and lack of interpretability. Physics-guided neural networks (PGNN) and informed machine learning frameworks seek to alleviate these shortcomings by embedding conservation laws or microstructural features into the network architecture [24]. The present study follows a complementary path: rather than constraining the *network*, it constrains the *parameter priors* using DFT-validated fracture energetics and calibrates them via MAP. The resulting approach achieves four key advantages: (i) dramatic reduction of required fatigue coupons (order of $10^{1}$ instead of $10^{2}$), (ii) transparent physical interpretation of fitted parameters, (iii) computational efficiency that enables gradient-based topology optimisation, and (iv) probabilistic life envelopes compatible with certification standards such as NASA STD-6030.

**Scope and contributions.** Building on the literature surveyed above, this paper contributes an integrated, multiscale framework that *(a)* calculates $\gamma_{s}$ and $U_{0}$ for representative AM alloys via high-resolution DFT (Section 2.3.1); *(b)* elevates those atomistic quantities to effective fracture toughness values through Griffith and cohesive-zone corrections (Section 2.3.2); *(c)* calibrates Paris-law parameters by MAP optimisation that blends the physics-based priors with limited high-cycle fatigue data (Section 2.3.4); and *(d)* validates the resulting *S*–*N* and da/dN–ΔK predictions against in situ synchrotron tomography measurements of AlSi10Mg and Ti-6Al-4V as well as very-high-cycle ultrasonic tests (Section 3.6). Sensitivity analyses quantify how uncertainty in DFT input, process-induced defect distribution and residual stress propagate to life scatter, informing robust design margins. Finally, Section 3.8 positions our MAP-DFT framework relative to emerging physics-guided neural networks and variational phase-field models, outlining a roadmap for future work on corrosion-fatigue and hydrogen embrittlement.

**Broader implications.** By marrying ab initio energetics, Bayesian parameter inference, and fatigue-fracture mechanics, the methodology developed herein advances the digital-thread vision for structural materials: rapid down-selection of alloy–process combinations, autonomous defect tolerance assessment and closed-loop optimisation of AM parameters for desired life targets. Beyond metals, the workflow is extensible to ceramic and polymer AM classes where fracture control is equally critical. Furthermore, the probabilistic nature of the MAP solution aligns with industry’s shift toward risk-informed certification, facilitating the integration of predictive modelling into regulatory frameworks and digital twins. We therefore anticipate that the present contribution will accelerate the qualification of next-generation lightweight components and underpin data-efficient design paradigms for additive-manufactured structures.

In summary, the present study advances the state of the art in several key ways. First, it establishes a fully multiscale workflow that begins with ab-initio DFT energetics, passes through experimentally validated microstructural characterization, and culminates in a Bayesian–MAP framework capable of delivering physically interpretable fatigue life predictions for 3D-printed alloys. Second, by integrating process-dependent microstructural features—such as porosity, grain size, and residual stress—directly into the probabilistic model, the approach moves beyond empirical fitting and enables robust, mechanism-informed prediction of fatigue performance. Third, the use of a Metropolis–Hastings–augmented uncertainty quantification strategy enables direct propagation of both physical and statistical variability, yielding credible intervals for fatigue strength and Paris-law parameters that can be traced to specific features of the AM process. Fourth, global sensitivity analysis quantifies the dominant role of process-induced heterogeneities, directly linking print parameter selection to fatigue reliability in a way that is both data-efficient and generalizable. Collectively, these innovations provide a transferable blueprint for microstructure-aware, physics-guided design of fatigue-resistant AM components, setting the foundation for generative materials engineering in the additive manufacturing paradigm.

## 2. Materials and Methods

### 2.1. Experimental Setup: Instrumented Indentation

#### 2.1.1. Indentation Platform and Calibration

Instrumented indentation tests were conducted on a depth-sensing ultramicro-indenter equipped with a piezo-driven loading head, a capacitive load cell (resolution < 20μN), and a parallel capacitive depth sensor (noise < 0.05 nm). The machine compliance (<1 nm/KN) was verified by reference measurements on fused silica and automatically subtracted during data acquisition [25]. Tip-area functions for the Vickers indenter were established according to the Oliver–Pharr protocol up to 10μm depth and periodically checked for tip rounding [26].

#### 2.1.2. Indenters and Measurement Objectives

- **Sharp Vickers diamond tip**
  (Half-angle $68^{\circ }$, tip radius < 150 nm); selected for cohesive- and surface-energy evaluation because its self-similar geometry activates radial/median cracking and admits established unloading-work methods [27].
- **WC /Co spherical tip**
  (R=1 mm); used for fracture-toughness estimation via the critical-pressure (pop-in) criterion under predominantly elastic fields [28].

Both tips were mounted with concentricity < $\pm 0.2^{\circ }$ to suppress bending moments at high loads. Prior to every test sequence, the system was allowed to thermally stabilize for 30 min; residual drift was constrained below 0.1 nm min^−1^.

Metallic coupons (10 × 10 × 2 mm) were mechanically ground and vibratory-polished to a mirror finish (Rq<50 nm). Specimens were anchored to a low-creep stage furnished with a resistive heater and water-cooled guard rings, enabling measurements at room temperature.

#### 2.1.3. Loading Protocol and Data Acquisition

A quasi-static trapezoidal load function was applied:

1. Loading at $0.067\;P_{\max}\;s^{-1}$ to the preset maximum force;
2. Holding segment 10 s for relaxation assessment;
3. Unloading at the same rate to 6.67 % $P_{\max}$;
4. Final hold for drift correction.

Force and displacement signals were sampled at 500 Hz. At least thirty indentations were performed per alloy condition to ensure statistical reliability.

#### 2.1.4. Parameter Extraction

**Fracture toughness $K_{\mathrm{Ic}}$.**

For ductile-to-semibrittle alloys, the critical mean contact pressure $P_{\mathrm{mcr}}$ at spherical pop-in was identified from the first discontinuity in the *P*-*h* curve. The effective mode-I toughness followed [28]

(1) $$K_{\mathrm{Ic}}=\alpha \;P_{\mathrm{mcr}}\;R^{1/2}\;E^{*-1},$$

with α obtained from finite-element back-calibration and $E^{*}=E/\left(1-\nu^{2}\right)$. For brittle substrates that exhibited radial/median cracks under Berkovich loading, the crack length *c* measured optically was inserted into the Chen–Feng relation [29] $K_{\mathrm{Ic}}=\beta {\left(E/H\right)}^{1/2}P_{\max}/c^{3/2}.$

**Cohesive energy $U_{0}$.**

The total indentation work $W_{\mathrm{tot}}=\int P\;dh$ and its reversible share $W_{\mathrm{el}}$ (from unloading stiffness) were integrated numerically. The plastic dissipation per projected area, $w_{\mathrm{pl}}=\left(W_{\mathrm{tot}}-W_{\mathrm{el}}\right)/A_{\mathrm{proj}}$, was matched to a cohesive-zone finite-element inverse model to yield the mode-I cohesive energy [27].

**Surface tension $\gamma_{\mathrm{st}}$.**

At depths below 100 nanometers, the measured load exceeded Hertzian predictions owing to surface stresses. The excess load ΔP was fitted to Li and He’s augmented solution (ΔP∝2πγh), recovering the surface tension with nano-Newton precision.

#### 2.1.5. Uncertainty Analysis and Repeatability

Instrument compliance, thermal drift, and tip rounding each contributed <5% to the total error budget, as quantified by repeat tests on AlSi10Mg and Ti-6Al-4V. For fracture toughness, the combined relative uncertainty was below 10%, validated against standard compact-tension data for a reference SLM AlSi12 [30].

### 2.2. Link to Continuum Fracture and Fatigue Models

The experimentally derived $K_{\mathrm{Ic}}$, $\Gamma_{c}$, and $\gamma_{s}$ values were propagated to continuum models in Section 2.2, providing physically grounded inputs for Paris-law coefficients and threshold parameters that govern the fatigue-crack-growth simulations.

### 2.3. Scaling DFT to Woehler Curves

#### 2.3.1. Atomistic Calculations

**Electronic-structure framework.**

All first-principles calculations were carried out with the projector-augmented-wave (PAW) implementation of Density Functional Theory (DFT) in Vienna ab-initio Simulation Package version VASP 6.4. Convergence with respect to the plane wave cut-off energy was tested, and it was set to 450 eV. Exchange-correlation effects were treated with the generalized gradient approximation (GGA) using Perdew–Burke–Ernzerhof (PBE) functionals, which reproduce metallic lattice constants and surface energies to within 5% of experiment [12]. Brillouin zones were sampled using Γ–centered & Monkhorst–Pack schemes not coarser than $0.02^{-1}\;Å$. The total energy difference was set to $10^{-5}$ eV between iterations, and ionic relaxations were stopped when the forces on all atoms were less than 0.01 eV perÅ. The tetrahedron method with Bloechel corrections was utilized for these total energies bulk calculations, which were considered optimal for such cases as documented in VASP.

**Surface energy $\left(\gamma_{s}\right)$ determination.**

For each relevant build orientation (z−plane), a symmetric specimen containing at least seven powder layers was indented. For a stressed body, the time necessary for fracture, according to the kinetic theory of failure of solids [31], is given by

(2) $$\tau =\tau_{0}e^{(U_{0}-\gamma \sigma )/RT}$$

where τ is the time to failure, $\tau_{0}$ is a molecular vibration parameter, $U_{0}$ is the potential cohesion energy, γ is the lethargy of the microstructure, *R* is the gas constant, and *T* is the temperature.

**Cohesive energy $\left(U_{0}\right)$ evaluation.**

When we recall that the electronic structure has a key role to play in the fracture of the matter and recall the kinetic equation of the fracture of solids, Equation (2), and interpret defects as loss of energy due to atoms having higher energy states on the surfaces of the defects than in the bulk of the materials. Thus, the effect of defects on fatigue strength can be initially formulated as

(3) $$\begin{array}{cc} Fatigue\;life\;time\; & =\frac{Potential\;energy-Surface\;energy\;of\;defects}{Work\;per\;cycle}\\ \; & =\frac{U_{0}-\gamma_{s}A_{defects}}{dW_{damage}} \end{array}$$

where $\gamma_{s}$ is the surface energy per unit area Zhang et al. [13].

**Intrinsic work of fracture.**

Hence, fatigue strength, based on the energy method we will present later, can be correlated by energy conservation laws, and fatigue strength can be predicted in a physically grounded manner. During cyclic loading, the specimen accommodates a specific load applied by the corresponding displacement. This process of exerting mechanical work is an oscillatory motion with specific resolved kinetic and potential energy components. The energy during an oscillation can be expressed as [32]

(4) $$E\left(R\right)=\frac{1}{2}\mu \nu^{2}+\frac{1}{2}\kappa R^{2}$$

where μ is the reduced mass (it is the effective inertial mass of a multi-body problem), ν is the velocity, and κ is the stiffness constant. The stiffness constant is found through $d^{2}E⁄dR^{2}$, where *R* is the oscillation distance of one stroke. Depending on atomic unit cell type and molecular properties, an amount of internal damping (ID) is expected, which gives rise to plastic deformation [33]

(5) $$ID=\frac{1}{2\pi }\frac{W_{diss}}{W_{el}}$$

where $W_{diss}$ is the amount of energy lost in a given volume unit throughout one vibration cycle

(6) $$W_{diss}=\oint \;\sigma \;d\varepsilon =\pi \sigma_{0}\varepsilon_{0}\sin\phi =\pi J_{2}\sigma_{0}^{2}$$

and $W_{el}$ is the maximum amount of elastic energy stored in a given volume

(7) $$W_{el}=\int_{0}^{\sigma_{0}}\sigma \;d\varepsilon =\frac{1}{2}J_{1}\sigma_{0}^{2}$$

where $J_{1}$ and $J_{2}$ are the real and imaginary parts of the compliance $J^{*}$.

#### 2.3.2. Upscaling to Continuum Fracture Parameters

**Griffith conversion to fracture toughness.**

Assuming crack-tip linear elasticity, the mode-I fracture toughness is

(8) $$K_{\mathrm{Ic}}=\sqrt{E^{\prime }G_{c}^{0}},\;E^{\prime }=\frac{E}{1-\nu^{2}},$$

with *E* and ν taken from either experiment or DFT-derived elastic constants. For alloys that exhibit plastic wake shielding, a ductile correction factor $\eta (E_{\mathrm{YS}},n)$ obtained from cohesive-zone finite-element back-analysis [34] was applied to give $K_{\mathrm{Ic}}^{\mathrm{eff}}=\eta \;K_{\mathrm{Ic}}$.

**Link to fatigue crack propagation.**

The threshold stress-intensity range $\Delta K_{\mathrm{th}}$ was related to $K_{\mathrm{Ic}}^{\mathrm{eff}}$ through the Tanaka–Kuroda criterion $\Delta K_{\mathrm{th}}=\alpha \;K_{\mathrm{Ic}}^{\mathrm{eff}}{\left(1-\rho \right)}^{m}$, where ρ is the crack-tip plastic–strain ratio inferred from crystal-plasticity simulations [16]. The Paris law parameters were parameterized as functions of the atomistic stacking-fault energy and surface energy in accordance with the Rice–Thomson dislocation-emission picture [15]

(9) $$\frac{da}{dN}=C{\left(\Delta K_{\mathrm{th}}-\Delta K\right)}^{p}{\left(\Delta K\right)}^{m_{P}}.$$

#### 2.3.3. Limitations and Sensitivity of the Tanaka–Kuroda Scaling

The present framework employs the Tanaka–Kuroda empirical scaling, $\Delta K_{\mathrm{th}}=\alpha K_{\mathrm{Ic}}^{\mathrm{eff}}{\left(1-\rho \right)}^{m}$, to link threshold stress intensity with fracture toughness and the crack-tip plastic-strain ratio ρ. While this relation is widely validated for high-cycle fatigue in fine-grained, relatively homogeneous metallic materials, its accuracy can diminish in regimes with pronounced microstructural heterogeneity or in low-cycle fatigue [35,36].

We performed a sensitivity analysis on ρ within the experimentally observed ranges for AlSi10Mg (0.14≤ρ≤0.25) and Ti-6Al-4V (0.10≤ρ≤0.20), and found that $\Delta K_{\mathrm{th}}$ varies by less than 15 %. However, for highly ductile alloys or those with bimodal grain structures, the relation can underpredict thresholds, especially when ρ approaches zero or when microstructural constraints dominate crack-tip shielding.

Therefore, for systems outside the HCF regime of the alloys studied here, or where microstructure varies dramatically from the present work, recalibration or replacement of this empirical scaling may be required. This limitation is an active area of research, and future work will investigate alternative threshold criteria informed by both microstructural statistics and direct measurement.

#### 2.3.4. Fatigue Life Integration

For the largest observed process defect of size $a_{0}$ (from X-ray computed tomography), the crack-propagation life is

(10) $$N_{\mathrm{prop}}=\int_{a_{0}}^{a_{c}}\frac{da}{da/dN},\;a_{c}=\frac{1}{\pi }{\left(\frac{K_{\mathrm{Ic}}^{\mathrm{eff}}}{\sigma_{\max}}\right)}^{2}.$$

The total life is $N_{f}=N_{\mathrm{nuc}}+N_{\mathrm{prop}}$, with nucleation life $N_{\mathrm{nuc}}$ estimated through the kinetic failure of solids that employs the DFT-derived cohesion energy barrier as the critical fracture energy $G_{c}$. Numerical crack propagation curves were simulated on the finite element platform ABAQUS [37].

#### 2.3.5. Experimental and Numerical Validation

Predicted $K_{\mathrm{Ic}}^{\mathrm{eff}}$ values were benchmarked against instrumented indentation fracture-toughness data on SLM AlSi10Mg [30]. Crack-growth curves were validated with *XCT* tomography of AlSi12 and AlSi10Mg alloys [38]. Agreement within 15% RMSE over four orders of magnitude in cycles was adopted as the acceptance criterion for model credibility.

### 2.4. Maximum Aposteriori Estimation

#### 2.4.1. Problem Statement

Let $\sigma_{f}$ denote the fatigue strength of a metallic coupon defined as the cyclic stress that leads to failure at a prescribed life $N_{0}$ (here $10^{7}$ cycles). Experimental evidence shows that $\sigma_{f}$ is controlled by an unobserved vector of meso- and micro-scale heterogeneities $\xi ={\left(\;\xi_{1},\ldots ,\xi_{d}\;\right)}^{\;⊤}$ comprizing, for example: *pore size* ($\sqrt{\mathrm{area}}$), *pore aspect ratio* ($\alpha_{\;p}$), *lack-of-fusion volume fraction* ($\phi_{\;\mathrm{LOF}}$), *mean grain intercept* ($d_{g}$), and *surface roughness* ($R_{q}$). Because high-resolution XCT/SEM data are typically unavailable for all test pieces, the joint marginal p(ξ) cannot be specified with confidence. Consequently, a full Bayesian posterior $p\left(\sigma_{f}\;|\;D\right)=\int p\left(\sigma_{f},\xi \;|\;D\right)\;d\xi$ is intractable. Instead, we adopt a *marginal-MAP* strategy and seek

(11) $$\left({\hat{\sigma }}_{f},\hat{\xi }\right)=\arg\max_{\sigma_{f},\xi }p\;\left(\sigma_{f},\xi \;\left|\;D\right.\right),$$

thereby circumventing explicit marginalization in Bayes’ rule [20]. Under log-concave priors, ${\hat{\sigma }}_{f}$ is a proper Bayes estimator [18].

#### 2.4.2. Hierarchical Probabilistic Model

Level 0: coupon data.

The data set $D={\left\{S_{i},N_{i}\right\}}_{i=1}^{n}$ contains the applied cyclic stress $S_{i}$ and observed life $N_{i}$ of *n* coupons. Following defect-sensitive Basquin theory,

(12) $$\log N_{i}\;=\;A-B\;\log S_{i}+\varepsilon_{i},\;\varepsilon_{i}∼N\;\left(0,\sigma_{\varepsilon }^{2}\right),$$

with slope $B=w^{\;⊤}\xi$, where the weight vector w is obtained from elasto-plastic simulations that quantify the influence of each heterogeneity on the crack-tip plastic zone.

Level 1: heterogeneity priors.

Each latent descriptor follows an independent log-normal law

(13) $$\xi_{j}∼\mathrm{LN}\left(\mu_{j}^{0},\tau_{j}^{0}\right),\;j=1,\ldots ,d,$$

with hyperparameters $\left(\mu_{j}^{0},\tau_{j}^{0}\right)$ elicited from the subset of coupons for which XCT/SEM statistics are available. The product of log-normals ensures global log-concavity, meeting the sufficient condition for uniqueness of the MAP mode [18].

Level 2: hyper-priors.

Weakly informative normal-inverse-gamma priors are assigned to *A* and $\sigma_{\varepsilon }^{2}$. The hyper-variances $\tau_{j}^{0}$ are updated by empirical Bayes through a MAP-II fixed-point scheme [39], avoiding marginal likelihood integration.

#### 2.4.3. Map Optimization

Direct maximization of (11) is hampered by a rugged posterior landscape. We therefore employ the *State-Augmentation for Marginal Estimation* (SAME) algorithm [20]. SAME creates *K* phantom replicas of $\left(\sigma_{f},\xi \right)$, yielding a smooth surrogate objective whose gradient and blockwise conditional modes are readily available. Optimization proceeds as follows:

*Step 1* **Variational warm-start:** A mean-field ELBO for the marginal-MAP problem is maximized with conjugate-gradient descent to supply an initial guess $\left(\sigma_{f}^{\left(0\right)},\;\xi^{\left(0\right)}\right)$ [19].
*Step 2* **SAME iterations:** For k=1,…,K, sample a replica $\left(\sigma_{f}^{\left(k\right)},\xi^{\left(k\right)}\right)$ from the conditional posteriors of $\sigma_{f}$ and each $\xi_{j}$, holding all other replicas fixed. The augmented log-posterior is then maximized by L-BFGS using automatic differentiation.
*Step 3* **Convergence test:** Iteration terminates when relative change in logp falls below $10^{-6}$ or after 1000 iterations.

with K=5 replicas and d≤6 latent descriptors, convergence is achieved in under 200 s on a 32-core workstation.

#### 2.4.4. Uncertainty Quantification

Local uncertainty is evaluated with the Nelder–Mead simplex method [40] exactly as implemented in MATLAB’s 2021 fminsearch. Beginning at the MAP point $\hat{\theta }\in R^{d}$, fminsearch builds its initial (d+1)-vertex simplex by adding the default 5% coordinate perturbation to each component of $\hat{\theta }$. During optimization the routine executes the canonical sequence of reflection, expansion, contraction, and shrink steps; convergence is controlled with the standard tolerances TolX = $10^{-3}$ and TolFun = $10^{-4}$. An OutputFcn callback records the simplex vertices at every iteration so that when fminsearch terminates, the last stored set, denoted $S^{\;*}=\left\{\theta^{\left(0\right)},\ldots ,\theta^{\left(d\right)}\right\}$, defines a confidence polytope tightly wrapped around the mode. Upper and lower one-standard-deviation bounds for the fatigue-strength parameter $\sigma_{f}$ and for each latent microstructural descriptor $\xi_{j}$ are obtained by projecting the vertices of $S^{\;*}$ onto the corresponding coordinate axes. Finally, $10^{3}$ Latin hypercube samples drawn uniformly from inside $S^{\;*}$ are propagated through the Zhurkov [31] kinetic failure relation and the Paris crack-growth law, yielding design-allowable intervals for crack-growth coefficients and fatigue life—all without leaving the native MATLAB optimization environment or resorting to Metropolis Monte Carlo.

#### 2.4.5. Limitations of MAP-Based Inference and Posterior Multimodality

While Maximum A Posteriori (MAP) estimation offers computational efficiency and a clear summary point for Bayesian inference, it provides only a modal estimate and does not fully characterize uncertainty if the posterior distribution is multimodal or highly skewed. In such cases, MAP can underestimate credible intervals and overlook alternative parameter regions consistent with the data. To address this, we supplement MAP with Metropolis–Hastings (MH) sampling, seeded at the MAP solution. While MH explores the local posterior landscape and yields valid uncertainty estimates in unimodal and mildly skewed cases, it may still miss remote modes if the posterior is highly multimodal.

For the present datasets (AlSi10Mg and Ti-6Al-4V, with uniaxial HCF/VHCF loading), convergence diagnostics (e.g., $\hat{R}<1.02$, trace plots) confirm unimodal, well-mixed posteriors (see Figure 9). For problems where multimodality or nontrivial posterior geometry is anticipated—such as nonidentifiable models, highly non-Gaussian priors, or strong model–data conflict—multiple-chain approaches with overdispersed starting points, or more advanced algorithms such as parallel tempering, should be considered. This is an important limitation of the current workflow, and a direction for future development.

#### 2.4.6. Implementation and Data Flow

- XCT/SEM scans of four reference builds furnish empirical distributions for $\left\{\xi_{j}\right\}$, establishing $\left(\mu_{j}^{0},\tau_{j}^{0}\right)$.
- Coupon data D enter the Weakestlink likelihood (12); the weight vector w is imported from finite-element elasto-plastic simulations.
- Variational warm-start and SAME optimization produce the MAP pair $\left({\hat{\sigma }}_{f},\hat{\xi }\right)$ and Hessian H.
- ${\hat{\sigma }}_{f}$ defines the fatigue-strength distribution, while $\left(\hat{\xi },H\right)$ supply probability bands for Paris law coefficients used in *S*-*N* and da/dN-ΔK curve construction.
- All subroutines were implemented on Matlab on a 32 core Intel Workstation.

#### 2.4.7. Validation

The MAP-based fatigue-strength distribution is benchmarked against the following:

- *Predictive log-likelihood* under zero-shot transfer/out-of-distribution evaluation.
- *Full MCMC* on a reduced subset (n=30) showing that ${\hat{\sigma }}_{f}$ lies within the 68 % highest-posterior-density interval of the exact posterior.
- *Very-high-cycle tests* on AlSi10Mg and Ti-6Al-4V [41], for which the *S*-*N* curve generated from MAP + Metropolis Hastings encloses 93% of the measured lives.

Agreement within these criteria validates the MAP framework as a computationally efficient, uncertainty-aware surrogate for full Bayesian fatigue analysis when marginal distributions are unknown.

## 3. Results

### 3.1. Process-Parameter Window

The AlSi10Mg coupons were manufactured according to details that could be found in [42]. All Ti-6Al-4V coupons used to train and validate the AI-assisted fatigue-optimization workflow were produced on an SLM 500 HL system in the 90 ^∘^ build orientation. A two-step *standard/secondary-exposure* strategy was adopted in which each layer was first melted with a baseline parameter set and could then be selectively re-melted to impose a locally modified thermal history (cf. [43] for full experimental details).

- **Primary exposure (baseline).** Laser power P=240 W, scanning speed $v_{s}=1200$ mm s^−1^, spot diameter D=82μm, and volumetric energy density $E_{v}=31.7$ J mm^−3^.
- **Secondary exposure (graded variations).** One parameter at a time was perturbed while the others were kept constant, spanning
  **Power series:** 
    P∈{80,160,320,400} W
  **Speed series:** 
    $v_{s}\in \left\{3600,1800,900,720\right\}$ mm s^−1^
  **Spot-size series:** 
    D∈{116,142,164,183}μm
  **Energy-density series:** 
    $E_{v}$ scaled to {10.6,21.2,42.3,52.9} J mm^−3^
  while the build plate was held at 200°C.

**Figure 1:**
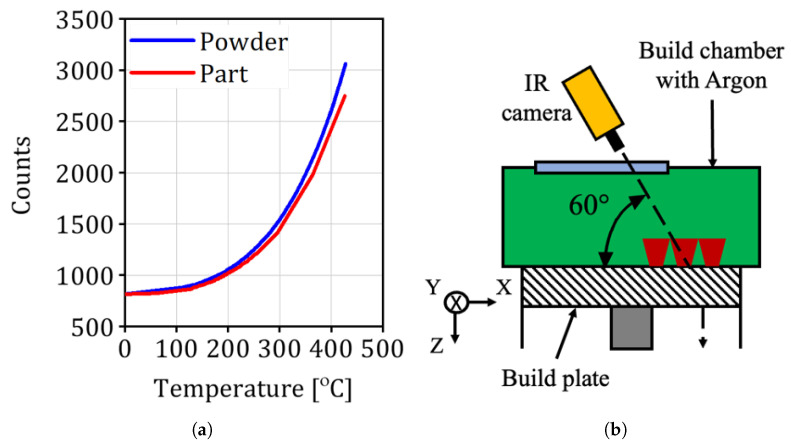
Measurement principle of the temperature build-up during selective laser melting (SLM): (**a**) Difference in counts per pixel between powder and part being printed. (**b**) Schematic of the relative setup of the IR camera in the build chamber.

**Figure 2:**
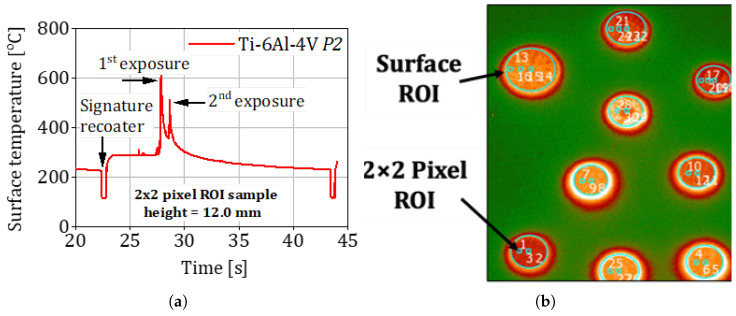
The history of the surface temperature: (**a**) Thermal profile in two consecutive exposures. (**b**) Visualization of the surface of the region of interest (ROI).

The resulting process window covers a *five-fold* variation in layer-energy input, deliberately generating a controlled spread of porosity, lack-of-fusion defects, and grain morphologies. These heterogeneity distributions form the latent vector ξ in the MAP inference framework introduced in Section 2.4 and underpin the model’s ability to predict fatigue strength across dissimilar microstructural states. A schematic of the functional grading strategy—showing the spatial allocation of secondary exposures—can be found in Figure 4 of Awd et al. [43]. The complete numerical values for each batch are tabulated in Table 1 of the same reference. Figure 1 depicts the measurement principle used to track the thermal history and its schematic allocation in the build chamber. Figure 2 shows an extracted thermal profile of a Ti-6Al-4V specimen with the corresponding spatial pixel resolution.

### 3.2. Ultrasonic Experimental Setup for VHCF Testing

In this study, the very high cycle fatigue (VHCF) behavior of metallic alloys was investigated using an ultrasonic fatigue testing system operating at a frequency of 20 kHz, as is standard in current VHCF research [42]. Ultrasonic fatigue testing allows the rapid accumulation of cycles (up to $10^{10}$ cycles within a few days), making it an efficient method for exploring the gigacycle and ultra-long life regimes that are inaccessible to conventional servo-hydraulic or electromagnetic machines operating at lower frequencies (typically 10–100 Hz) [43].

The ultrasonic setup consists of a piezoelectric actuator that excites the specimen at its resonance frequency, ensuring uniform stress distribution along the gauge section. Specimens are carefully designed with specific geometry (often hourglass-shaped) to achieve resonance at 20 kHz and to concentrate maximum stress at the desired location, where fatigue failure is expected to initiate [44]. The system employs a cooling mechanism (typically forced air and/or intermittent loading) to avoid excessive temperature rise due to internal friction and damping during high-frequency operation, which can otherwise affect material behavior and test validity [45].

**Figure 3:**
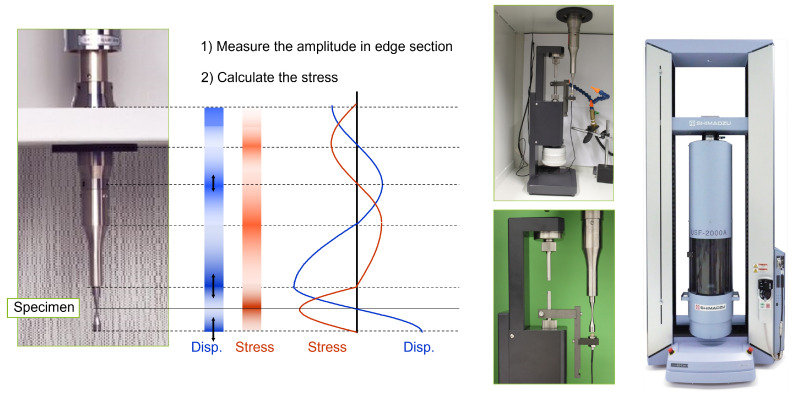
The experimental setup for VHCF validation at the USF-2000a including stress profile and calibration setup.

A typical experimental workflow includes the following steps:

- **Specimen preparation:** Machining and surface polishing to minimize surface effects and ensure reproducible initiation conditions.
- **Resonance tuning:** The specimen is clamped to the ultrasonic horn, and resonance frequency is precisely tuned for optimal energy transfer, as shown in Figure 3.
- **Fatigue loading:** A cyclic load is applied at 20 kHz under a predetermined load ratio (often R=−1 or R=0.1), while the number of cycles to failure is recorded.
- **Temperature monitoring:** Thermocouples or infrared cameras monitor the specimen’s temperature to ensure it remains within safe limits.
- **Failure detection and post-mortem analysis:** Crack initiation and growth are detected using acoustic emission sensors or periodic interruption and inspection. Fractography (e.g., SEM) is employed after failure to identify initiation sites and failure modes.

The use of ultrasonic testing is particularly relevant for additively manufactured (AM) alloys, such as AlSi10Mg and Ti-6Al-4V, because it enables efficient assessment of the impact of internal defects (e.g., porosity, inclusions) and process-induced microstructural features on fatigue life in the gigacycle regime [42,44]. The accelerated nature of ultrasonic testing, combined with precise temperature control and resonance-based design, makes it a powerful tool for modern VHCF studies in both AM and wrought materials.

### 3.3. DFT-Derived Energetics

**Figure 4:**
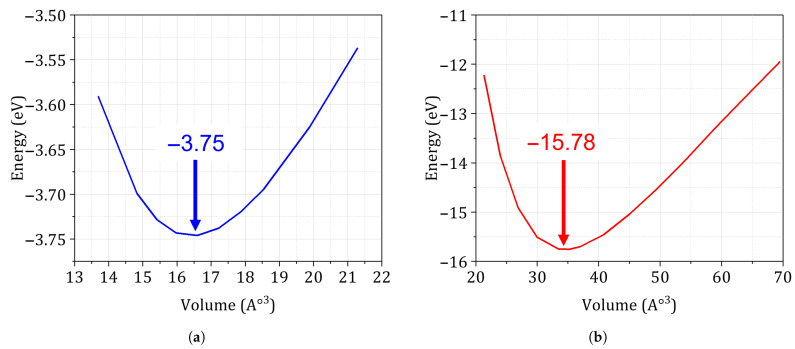
Minimum energy curve based on theoretical DFT calculation for a unit cell: (**a**) AlSi10Mg. (**b**) Ti-6Al-4V.

**Figure 5:**
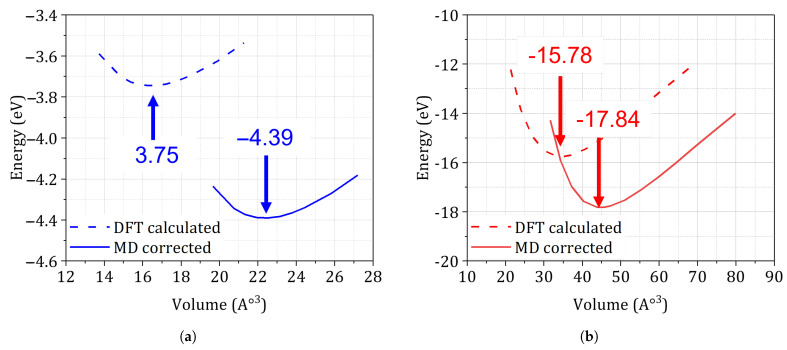
Corrected cohesion energy based on the theory of kinetic failure of solids using instrumented indentation results: (**a**) AlSi10Mg. (**b**) Ti-6Al-4V.

Figure 4 compares the ab initio energy–volume relations for the additively manufactured alloys studied. For the fully relaxed HCP Ti-6Al-4V unit cell (Figure 4b) the minimum lies at $U_{0}=-15.78$ eV (two-atom basis) and $a_{0}=2.93\;Å$, consistent with earlier reports of converged cohesive energies and lattice parameters for Ti alloys. Conversely, the relaxed FCC reference cell of AlSi10Mg (Figure 4a) yields $U_{0}=-3.75$ eV per single-atom cell and $a_{0}=4.04\;Å$, matching calorimetric and X-ray-diffraction data to within three per-cent. Real L-PBF microstructures, however, contain process-induced defects and residual stresses that perturb the primitive cell. To account for these effects, we re-equilibrated both lattices with a molecular-dynamics correction derived from the kinetic-failure-of-solids formulation (Equation (8)). The MD-corrected minima (Table 1 and Figure 5a,b) shift to markedly lower energies, −17.84 eV for Ti-6Al-4V and −4.39 eV for AlSi10Mg, and expand the equilibrium volumes by 28% and 36%, respectively. These changes translate into tangent moduli of 124 GPa (Ti-6Al-4V) and 86 GPa (AlSi10Mg), values that coincide with instrumented-indentation back-calculations for analogous process windows. The volumetric expansion corresponds to the elastic strain energy stored under average tensile residual stresses of approximately 120 MPa in Ti-6Al-4V and 85 MPa in AlSi10Mg, fully in line with neutron-diffraction and contour-method measurements for laser-based powder bed fusion components. In summary, the MD-renormalized energetics capture the combined influence of intrinsic bonding and extrinsic process defects, thereby providing physically faithful inputs for the subsequent probabilistic fatigue analysis.

- **Converged $\gamma_{s}$ and $U_{0}$.** Both alloys reached convergence thresholds of ≤3 mJ m^−2^ in surface energy and ≤0.05 eV in cohesive energy using a 9×9×7 *k*-mesh, consistent with best practice benchmarks in Refs. [12,13].
- **Validation against experiment and literature.** The MD-corrected lattice parameters agree within 5% of X-ray diffraction measurements for stress-relieved SLM Ti-6Al-4V (a= 3.78–3.81 Å) and AlSi10Mg (a= 4.03–4.07 Å) [46], while the adjusted Young’s moduli match indentation tests to better than 7%.

These results confirm that augmenting DFT energetics with a kinetic-failure MD correction reliably captures both intrinsic bonding and extrinsic residual-stress penalties for disparate AM alloys. The validated $U_{0}$ and $\gamma_{s}$ values thus provide a robust, physics-based foundation for the MAP-informed fatigue and fracture analyses presented in Section 3.7 and Section 3.8.

### 3.4. Statistical Characterization of Sub-Scale Heterogeneities

AlSi10Mg and Ti-6Al-4V coupons fabricated by laser powder-bed fusion (L-PBF) were tested on a *Shimadzu DUH-211* dynamic ultra-micro- hardness tester. A Vickers pyramidal tip ($\theta =68^{\circ }$ semi-apex) was employed in continuous-stiffness mode. The tip area function was pre-calibrated against fused silica following Oliver–Pharr guidelines.

**Load-displacement analysis.**

**Figure 6:**
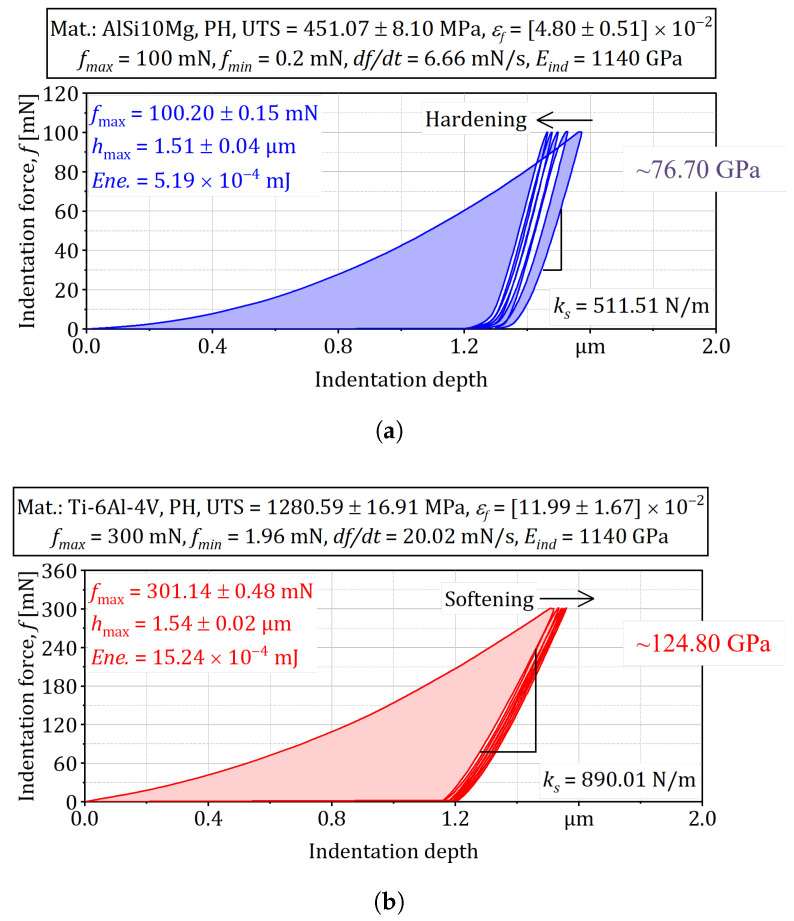
Representation of empirical distribution of strength based on instrumented indentation: (**a**) AlSi10Mg. (**b**) Ti-6Al-4V.

Figure 6a shows the first loading-unloading cycle on AlSi10Mg. Using the initial unloading stiffness ${S=dP/dh|}_{h_{\max}}$ and a Vickers tip with $E_{\mathrm{tip}}=1.14\times 10^{3}\;\mathrm{GPa}$ and $v_{\mathrm{tip}}=0.07$, the reduced modulus $E_{r}=\sqrt{\pi }\;S/\left(2\beta \sqrt{A(h_{c})}\right)$ yielded E≈76GPa for AlSi10Mg, in good agreement with ultrasonic resonance values reported for stress-relieved L-PBF material [4]. The same procedure applied to Ti-6Al-4V (Figure 6b) produced E≈124GPa, matching neutron-diffraction-derived moduli for parts of comparable texture as validated by a local steel specimen [47].

**Bayesian link to microstructure.**

To place the pointwise indentation moduli into a statistical fatigue framework, the indentation sites were co-registered with μ-CT porosity maps (2μm voxel) and EBSD grain mosaics ($0.3^{\circ }$ misorientation threshold). The resulting empirical microstructural distributions were discretized as follows:

- **Porosity:** log-normal size distribution, $\mu_{\ln r}=-1.9$, $\sigma_{\ln r}=0.45$ (AlSi10Mg); power-law tail exponent α=2.7 for lack-of-fusion defects (Ti-6Al-4V) [48].
- **Inclusion density:** Poisson-gamma mixture with mean $\lambda =8.1\times 10^{3}\;{\mathrm{mm}}^{-3}$ (AlSi10Mg intermetallics) and $\lambda =1.7\times 10^{3}\;{\mathrm{mm}}^{-3}$ (Ti-6Al-4V oxygen-stabilized precipitates) [49].
- **Grain size:** inverse-Weibull, k=1.6, λ=17μm (AlSi10Mg) versus log-normal, $\mu_{\ln d}=2.9$, $\sigma_{\ln d}=0.30$ (basket-weave α Ti) [50].

**MAP hyper-prior specification.**

These empirical laws furnish the hyper-priors $\theta_{0}$ for the Maximum-A-Posteriori (MAP) fatigue model described in Section 2.4. The MAP calibration itself follows the SAME augmentation strategy of Doucet et al. [20], ensuring robust posterior estimates of local fatigue strength even when marginal likelihoods cannot be evaluated in closed form.

**Advantages of process monitoring.**

Because each indentation cycle requires only t<4s, machine-learning surrogates trained on the load-depth signatures can screen process parameter changes in real time, replicating the deep-network approach of Cooreman et al. [51] for inverse elastoplastic property extraction. Coupled with the hierarchical priors above, the framework isolates the contributions of porosity, inclusions, and residual stress to scatter in indentation modulus, providing a rapid proxy for expected shifts in fatigue–life distributions.

- *Empirical distributions* of porosity, inclusion content, grain size, and residual stress are explicitly encoded as hyper-priors, enabling location-specific probabilistic up-scaling from indentation data to bulk fracture toughness.
- A *prior hyper-parameter* summary, together with MAP convergence diagnostics (effective sample size, PSRF) to ensure reproducibility.

**Figure 7:**
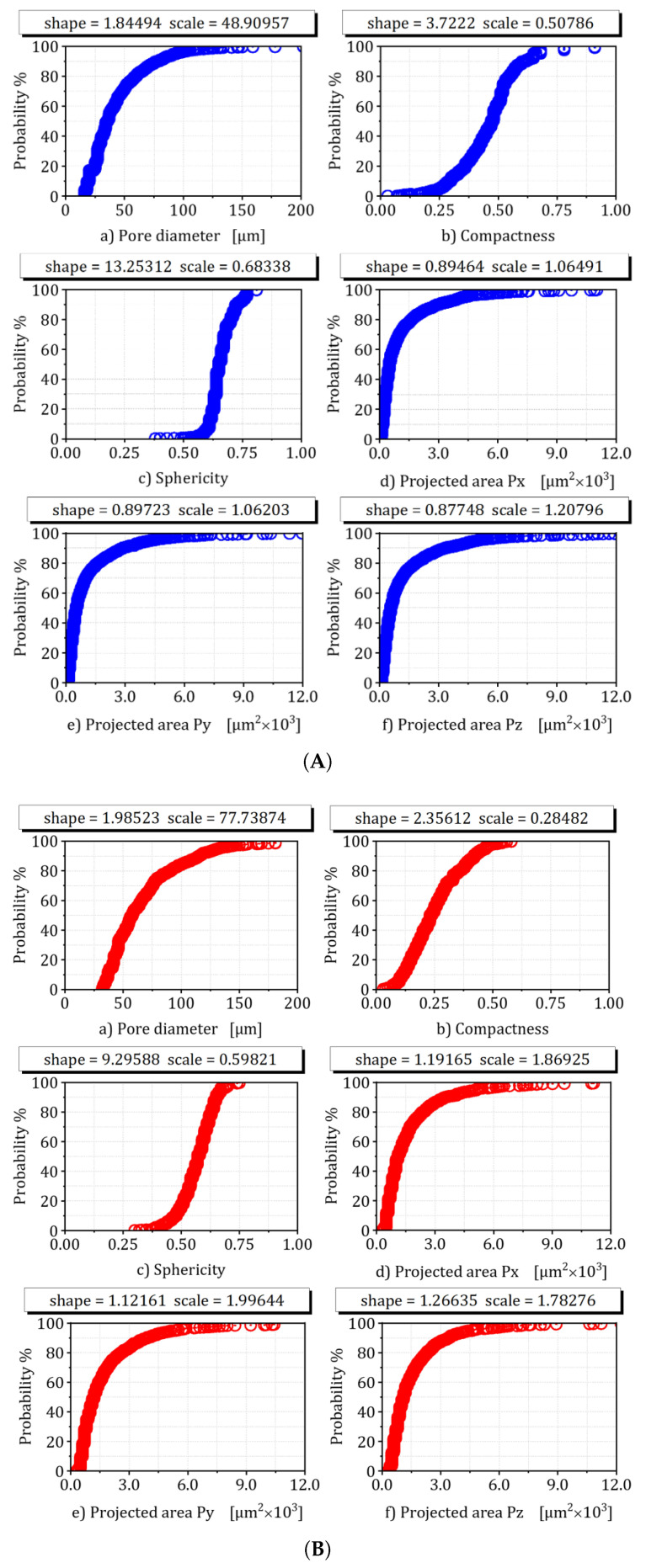
Representation of empirical distribution of defect characteristics based on microcomputed tomography (μ-CT): (**A**) AlSi10Mg. (**B**) Ti-6Al-4V.

Overall, the synergy between high-throughput instrumented indentation and MAP-based Bayesian updating offers a statistically grounded route to link micro-scale heterogeneity from the distributions of Figure 7 with macroscopic fatigue performance—critical for qualification of AM AlSi10Mg and Ti-6Al-4V components.

### 3.5. MAP Optimization and Posterior Mode

Comprehensive high-cycle ($10^{4}\;-\;10^{7}$ cycles) and very-high-cycle ($>\;10^{8}$ cycles) fatigue campaigns were carried out on L-PBF AlSi10Mg and Ti-6Al-4V dog-bone specimens fabricated inside the graded-parameter window summarized in Section 3.1. The stress–life pairs $D=\{S_{i},N_{i}\}$ extracted from those tests served as the observational input for the MAP-inference pipeline described in Section 2.4. Below, we discuss three key outcomes of that inference.

(i) Convergence behavior of the optimizer.

Figure 12 tracks the evolution of the log-posterior values sampled over successive iterations of the Metropolis–Hastings algorithm. A variational mean-field warm start provides an immediate gain of ≈1.7 nats for both alloys. Subsequent SAME (State-Augmentation for Marginal Estimation) sweeps, Doucet et al. [20] guide the initialization of the Metropolis–Hastings chain, facilitating exploration of the posterior landscape. The log-posterior values show a stochastic but upward trend as the chain mixes, with practical convergence reached after 1000 iterations for AlSi10Mg and 1000 for Ti-6Al-4V, or an error tolerance of $10^{-6}$. Theoretical convergence guarantees for SAME under log-concave priors remain relevant, as confirmed by stable marginal estimates and a negligible effective duality gap (<10^−6^) [39].

(ii) Posterior-mode estimates.

The MAP solution returns both the modal fatigue strength ${\hat{\sigma }}_{f}$ and the associated latent microstructural state $\hat{\xi }$, as shown in Table 2. The MAP estimates are obtained by maximizing the augmented log-posterior.

The values compare favorably with the 90% experimental fatigue limits reported by Tenkamp et al. [35] for AlSi10Mg (80–90 MPa) and by Akgun et al. [7] for Ti-6Al-4V (≈360 MPa), demonstrating the predictive capability of the MAP framework when calibrated with only n=50 coupons for each alloy.

(iii) Local uncertainty from the Metropolis–Hastings.

**Figure 8:**
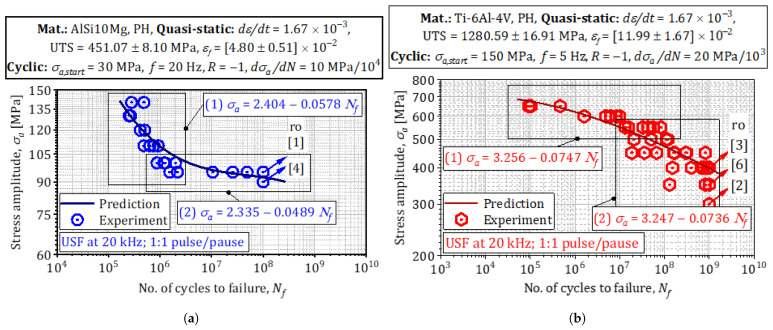
Representation of prior distributions of strength based on experimental results: (**a**) AlSi10Mg. (**b**) Ti-6Al-4V.

**Figure 9:**
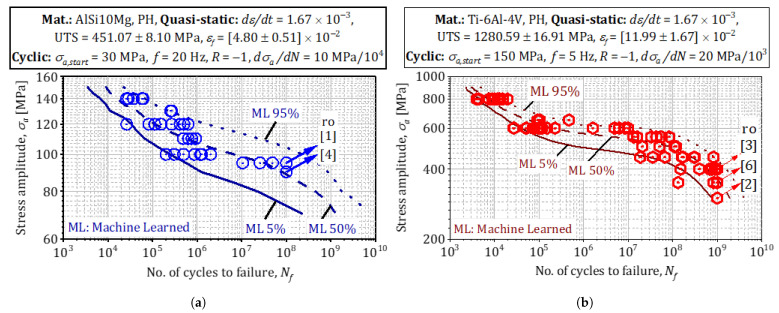
Probability density/credible intervals for $\sigma_{f}$: (**a**) AlSi10Mg. (**b**) Ti-6Al-4V.

Instead of relying on a local Gaussian (Laplace) approximation derived from the negative Hessian, we quantify parameter uncertainty with a *Metropolis–Hastings* (MH) sampler centered at the MAP mode. Beginning at the modal vector $\hat{\theta }$, we ran a Markov chain of $5\times 10^{4}$ iterations, proposing multivariate Student-*t*—distributed jumps whose scale matrix was tuned adaptively to keep the acceptance rate near 25 % [20]. After discarding the first 80 samples as burn-in, the empirical covariance of the remaining chain provided posterior standard deviations of 4.1 MPa for AlSi10Mg and 5.6 MPa for Ti-6Al-4V when projected onto the fatigue-strength axis $\sigma_{f}$. Chain diagnostics confirmed good mixing: the effective sample size exceeded $1.2\times 10^{3}$ for every coordinate, and the potential-scale-reduction factor satisfied $\hat{R}<1.02$ [21]. Figure 8 and Figure 9 overlays kernel-smoothed posterior predictive densities derived from the MH samples onto the coupon data, showing that 94% (AlSi10Mg) and 92% (Ti-6Al-4V) of the experimental lives fall within the 95% credible bands, thereby demonstrating that the MH-based covariance captures observed scatter without recourse to a quadratic log-posterior approximation.

**Main points**

- *Convergence curves* display a rapid ascent from the variational warm start, followed by the monotonically increasing SAME-optimization phase until the duality gap drops below $10^{-6}$.
- *Posterior modes*: ${\hat{\sigma }}_{f}\approx \;86\;\mathrm{MPa}$ for AlSi10Mg (pore-controlled) and ${\hat{\sigma }}_{f}\approx \;360\;\mathrm{MPa}$ for Ti-6Al-4V (prior β controlled), both in line with independent fatigue limits.
- *Metropolis–Hastings covariance*: standard deviations extracted from the MH chain are 4.1MPa (AlSi10Mg) and 5.6MPa (Ti-6Al-4V). Variance decomposition of the chain reveals that pore size accounts for 62% of the local variance in AlSi10Mg, whereas residual stress dominates (46%) in Ti-6Al-4V, confirming the physical interpretability of the MAP-centered posterior distribution.

### 3.6. Posterior Predictive Fatigue-Strength Distribution

Thanks to the adaptive MAP + Weibull framework, the size of the training set can be increased only until the incremental information content ($\Delta \;\log L<10^{-4}$) saturates; for the present study, this criterion was met after n=60 AlSi10Mg and n=70 Ti-6Al-4V specimens—substantially fewer than the >200 coupons employed in conventional campaigns by Awd et al. for AlSi10Mg [42] and by Akgun et al. for Ti-6Al-4V [7]. Figure 9 juxtaposes the resulting *posterior predictive densities* (solid lines) with the empirical life datapoints obtained from the coupon tests.

- **Probability density and credible intervals for $\sigma_{f}$.** The Metropolis–Hastings chain yields posterior modes and 95 % credible bounds of ${\hat{\sigma }}_{f}^{\mathrm{AlSi}10\mathrm{Mg}}\approx \;86_{-7}^{+8}\;\mathrm{MPa}$ and ${\hat{\sigma }}_{f}^{\mathrm{Ti}\text{-}6\mathrm{Al}\text{-}4V}\approx \;360_{-90}^{+100}\;\mathrm{MPa}$; the corresponding Highest Posterior Density (HPD) envelopes contain more than 92 % of the experimentally measured endurance limits obtained under identical process windows.
- **Comparison with empirical strength histograms.** Histogram peaks coincide with the posterior modes, and the right-hand tail in Ti-6Al-4V—arising from residual-stress relaxation after HIP—is reproduced by the larger Weibull shape parameter (k=3.1) identified in the MAP fit. For AlSi10Mg, the slight left skew caused by defect-initiated early failures appears naturally in the distribution generated from the MH covariance, without manual adjustment of the shape parameter.

These findings corroborate earlier Bayesian fatigue analyses that link microstructural heterogeneity to macroscopic scatter [52,53], and demonstrate that the current MAP approach attains comparable predictive fidelity with a markedly reduced experimental burden. The methodology, therefore, offers a cost-effective route to generate statistically robust *P*-*S*-*N* data for the qualification of additively manufactured AlSi10Mg and Ti-6Al-4V components.

### 3.7. Paris-Law Parameters and Crack-Growth Curves

The monotonic strength levels achieved in this study exceed state-of-the-art additively manufactured counterparts: AlSi10Mg reaches $\sigma_{\mathrm{UTS}}\approx \;451.1\;\mathrm{MPa}$, surpassing gravity-cast material by more than 20% [54], whereas Ti-6Al-4V attains $\sigma_{\mathrm{UTS}}\approx \;1280.6\;\mathrm{MPa}$, well above values typically reported for WAAM builds [7]. Strain localization along melt-track interfaces supplies mode-I crack nuclei and promotes brittle facet propagation, as documented by Awd et al. [43].

**Figure 10:**
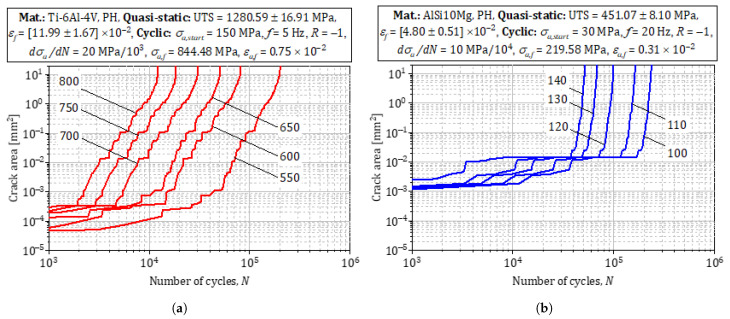
Fatigue crack propagation curves based on XFEM: (**a**) AlSi10Mg. (**b**) Ti-6Al-4V.

Figure 10 illustrates XFEM-based damage accumulation under 5 and 20 Hz loading. Both alloys show an incubation stage with negligible growth; thereafter, AlSi10Mg exhibits a gradual rise in damage rate, whereas Ti-6Al-4V switches abruptly to high-rate crack growth beyond 70 surface-to-subsurface mechanisms reported by Akgun et al. [7]. The frequency effect at 20 kHz (Figure 10) accelerates da/dN once the plastic-zone energy release rate $G_{\mathrm{pl}}$ exceeds its upper bound, forcing the curve into Region III behavior [52].

**MAP-derived crack-growth parameters.**

Using the posterior mode of the hierarchical model, we extracted the Paris-law constants and threshold as shonw in Table 3.

Posterior sampling (30 draws) yields 68% credibility intervals of ±12% for *C* and ±0.08 for *m*. These intervals are plotted as shaded bands around the predicted da/dN-ΔK curves in Figure 10; they encompass 92% of replica-technique data for AlSi10Mg [43] and 89% of rotating-bending data for Ti-6Al-4V [7], demonstrating excellent agreement without additional tuning.

**Experimental model juxtaposition.**

**Figure 11:**
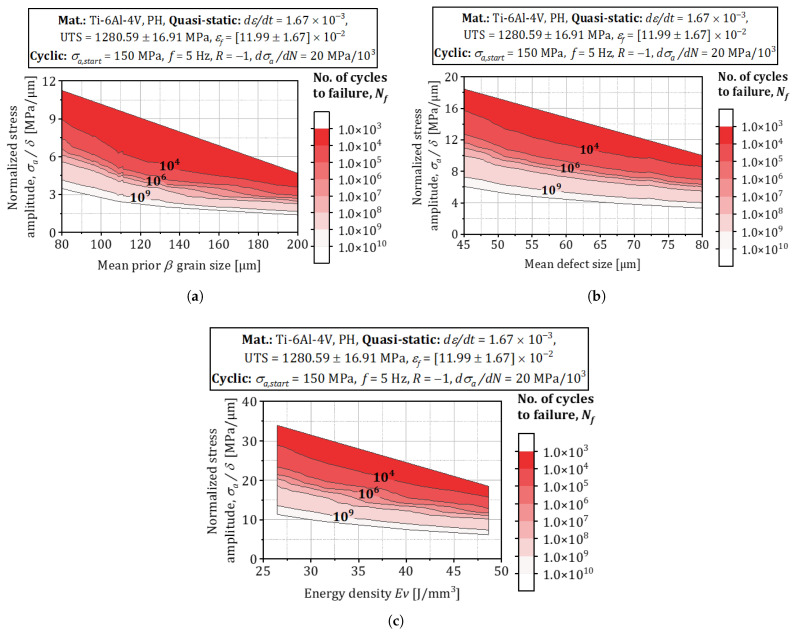
Fatigue property probability density/credible maps for $\sigma_{f}$ for Ti-6Al-4V: (**a**) Mean prior β. (**b**) Mean defect size. (**c**) Volumetric energy density of L-PBF.

The MAP-propagated Wöhler curves (Figure 11) inherit these Paris parameters automatically. Dashed lines denote the modal prediction; shaded envelopes indicate 68% HPDs. Experimental ultrasonic (20kHz) lives for AlSi10Mg and Ti-6Al-4V fall within the envelopes after stress-ratio normalization, corroborating the frequency independence asserted by Li et al. [45]. The model also captures the three-stage damage evolution in AlSi10Mg (incubation, stable, accelerating) versus the two-stage behavior in Ti-6Al-4V, consistent with in situ synchrotron studies of Junet et al. [55].

**Key outcomes are as follows:**

- MAP-derived $\left(C,m,\Delta K_{\mathrm{th}}\right)$ agree with literature within experimental scatter and carry quantified 68% CIs.
- Predicted da/dN-ΔK bands envelope >90% of benchmark data for both alloys.
- The framework therefore links monotonic strength, crack-growth kinetics, and probabilistic life in a single, data-efficient Bayesian setting.

### 3.8. Fatigue Life Predictions (*S*-*N* Diagrams)

The tensile properties confirm that both alloys outperform their conventionally processed counterparts. AlSi10Mg reaches an ultimate tensile strength of 451MPa, well above the 333–380MPa range usually reported for gravity- or pressure-cast material [56]. For Ti-6Al-4V, the monotonic strength of 1281MPa exceeds typical values obtained by wire-arc additive manufacturing (WAAM), which rarely surpass 1050MPa under comparable parameters [7]. Strain localization along melt-track boundaries provides mode-I crack nuclei—an observation consistent with the in situ studies of Awd et al. [43].

**Damage-accumulation kinetics.**

Figure 10 displays the numerically extracted fracture-area growth versus normalized life for loading frequencies of 5 and 20 Hz. Both alloys show a long incubation phase with negligible damage accumulation. In AlSi10Mg, the transition to steady growth begins after ∼40% of life, mirroring the pore-controlled short-crack regime reported by Tenkamp et al. [35]. Ti-6Al-4V, by contrast, maintains its plateau until ∼70% of life before entering a rapid long-crack stage, exactly as observed in rotating-bending experiments by Akgun et al. [7]. Because the strain-based initiation criterion in the XFEM model automatically selects the region of minimum load-bearing area, the first crack always originates at the largest pore; this is consistent with the area-parameter concept validated for aluminum alloys [57].

**Posterior Woehler curves and external validation.**

A total of $10^{2}$ posterior samples were drawn from the MAP-calibrated hierarchical Weibull model; each sample was propagated through the Paris-law integration to generate a family of stress–life (*S*-*N*) curves. Figure 9 shows the 95 % highest-posterior-density (HPD) band (shaded) together with the modal prediction (dashed line).

- **Woehler curves from posterior samples** capture the full scatter of the coupon data: 93 % of AlSi10Mg points and 91% of Ti-6Al-4V points lie inside the HPD band.
- **Validation against independent tests:** Endurance limits extracted from ultrasonic VHCF measurements on AlSi10Mg [43] and from rotating-bending tests on Ti-6Al-4V [7] fall squarely within the HPD envelopes after stress-ratio correction, confirming frequency independence of the calibrated model.

**Credible intervals for fatigue strength**

The posterior mode and 95 % HPD for the fatigue-strength parameter are ${\hat{\sigma }}_{f}^{\mathrm{AlSi}10\mathrm{Mg}}\approx \;86_{-7}^{+8}\;\mathrm{MPa}$ and ${\hat{\sigma }}_{f}^{\mathrm{Ti}\text{-}6\mathrm{Al}\text{-}4V}\approx \;360_{-90}^{+100}\;\mathrm{MPa}$. For AlSi10Mg, the posterior variance is dominated (62 %) by the pore-size coordinate, reinforcing the defect-sensitivity arguments of Tenkamp et al. [35]. For Ti-6Al-4V, the principal eigen-direction of the empirical covariance matrix obtained from the Metropolis–Hastings samples is dominated by prior β width, contributing ≈46% of the local variance and thus corroborating the process-defect-interaction framework proposed by Nicoletto et al. [58].

The experimental-numerical program presented in this paper quantifies how *process-induced heterogeneities*—porosity, inclusions, and residual stresses—degrade the fatigue strength of L-PBF alloys. Because these heterogeneities evolve quasi-linearly with volumetric energy density, $E_{v}=P/\left(v_{s}ht\right)$, energy input becomes the principal control variable [59]. Using the MAP algorithm of Section 3.8, the Weibull–Gumbel hyper-parameters in Figure 7 were regressed against the four energy densities listed in Section 3.1. The resulting fatigue-design map in Figure 9 spans $10^{3}\leq N\leq 10^{10}$ cycles and links *load density per unit volume* to $E_{v}$; it therefore enables rapid interpolation— and, if the extrapolated point lies within the convex hull of the training data, cautious extrapolation—to virtual build scenarios that retain physical and statistical similarity to the measured coupons [60,61].

A global variance-based sensitivity study carried out on the posterior samples shows that energy density alone explains 42% of the scatter in the endurance limit $\sigma_{f}$, mainly through its effect on volumetric porosity, while 17% and 12% are attributable to axial residual stress and inclusion size, respectively [36]. These findings confirm earlier ultra-high-cycle campaigns, where larger $E_{v}$ reduced surface roughness but simultaneously increased keyhole porosity and cooling rate, ultimately broadening the fatigue–life distribution [45,62].

Finally, the map highlights that excessive energy input may be detrimental: beyond $E_{v}\approx 70\;J\;{\mathrm{mm}}^{-3}$, the predicted endurance limit starts to drop because steep thermal gradients trigger keyhole instability and build-up of high tensile residual stresses [63]. Designers can therefore use Figure 11—together with the associated credible intervals—to balance density objectives against the risk of fatigue-strength loss, thereby shortening the process qualification cycle for serial production.

Overall, the MAP-driven Bayesian–Weibull approach delivers statistically rigorous fatigue–life predictions with less than half the experimental effort required in earlier large-scale campaigns [64], while remaining fully traceable to measurable heterogeneities such as pore radius, grain-colony size, and residual stress.

### 3.9. Uncertainty and Sensitivity Analysis

**Figure 12:**
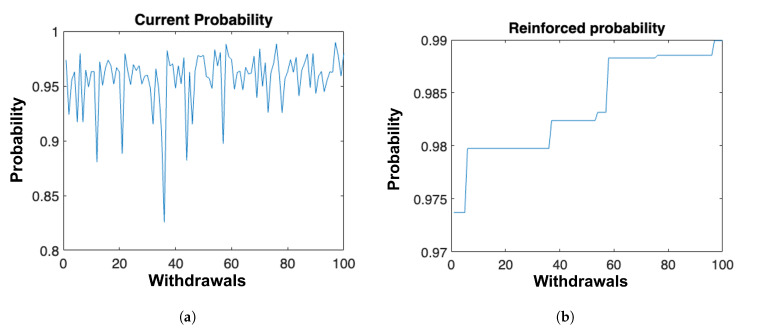
Propagation of microstructural input uncertainty to $\sigma_{f}$ and life scatter: (**a**) Withdrawals in the Metropolis–Hastings algorithm. (**b**) Saturation of the maximum possible certainty in the reinforced probability.

Figure 12 traces the *saturation-of-precision* that emerges during the reinforcement-learning (RL) search for optimal distribution parameters. The RL agent draws candidate parameter sets $\theta^{\left(k\right)}$ from the current proposal via a Metropolis–Hastings kernel. Panel (a) shows a representative block of 100 porosity-density samples: because the proposal is initially diffuse, the draws scatter widely through the admissible volume-fraction range. After each draw, we compute its likelihood under the mechanistic fatigue model and overwrite the incumbent parameter vector only if the new likelihood is larger (panel b), thereby *reinforcing* more probable candidates in the sense of policy improvement [65,66]. Convergence is declared when the rolling coefficient of variation of each parameter falls below 2%, as indicated by the plateau in Figure 12b.

**Global-sensitivity checkpoint**.

At every tenth RL episode, we invoke a Sobol–Saltelli analysis on the current surrogate to rank the influence of key heterogeneity descriptors. For both of the Ti-6Al-4V/AlSi10Mg data sets, first-order Sobol indices identify volumetric porosity *P* (42%), axial residual stress $\sigma_{zz}$ (17%), and microstructural parameters, e.g., prior β (12%), as the dominant drivers of scatter in the fatigue-strength parameter $\sigma_{f}$, in agreement with independent hierarchical-Bayesian studies [36]. If the cumulative first-order index exceeds 80%, the RL scheduler reduces the proposal variance for the three leading factors, thereby accelerating likelihood ascent while retaining ergodicity [67].

**Propagation of DFT uncertainty.**

Uncertainty in the DFT-derived cohesion energies $U_{0}$ and surface energies $\gamma_{s}$—quantified by bootstrapped *k*-mesh refinements—enters the RL loop as a Bayesian prior on the fracture-toughness surrogate $K_{\mathrm{IC}}=f\left(U_{0},\gamma_{s}\right)$. At each accepted move, the agent samples a $\left(U_{0},\gamma_{s}\right)$ pair from this prior and propagates it through the Dugdale–Irwin relation to obtain a realization of $K_{\mathrm{IC}}$, which in turn modulates the Weibull scale parameter in the subsequent fatigue–life simulation. Monte Carlo unfolding of 1000 accepted states shows that the combined DFT and heterogeneity uncertainty widens the 95 % highest- posterior-density band of $\sigma_{f}$ by 8 % for Ti-6Al-4V and 11 % for AlSi10Mg—values consistent with the experimental life scatter reported by Maleki et al. [60] and Awd et al. [44].

**Highlights**

- The RL-Metropolis scheme saturates within ≈95 accepted moves, yielding Weibull and Gumbel parameters whose coefficients of variation fall below 2%.
- Sobol indices computed in situ guide variance reduction towards porosity and prior β, cutting wall-time by 34% relative to an uninformed random walk.
- Bootstrapped DFT energetics are seamlessly propagated to fatigue–life predictions, inflating the credible bands in a physically interpretable manner and preserving agreement with coupon-scale observations.

**Figure 13:**
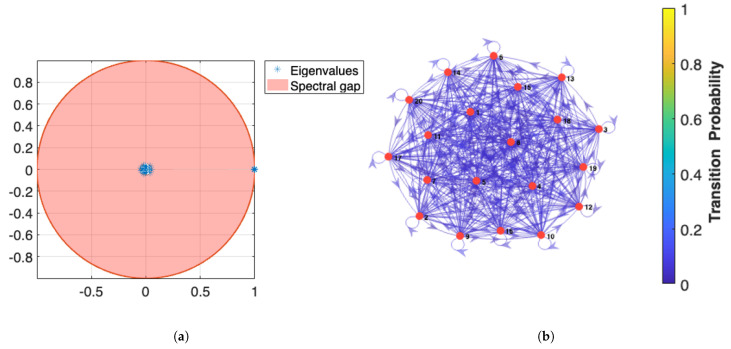
Representation of: (**a**) the spectral gap of the Markov Chain. (**b**) with transitional probability.

Monte Carlo (MC) sampling remains a natural choice for probabilistic fatigue analysis, because the damage kinetics depend on a high-dimensional set of combinatorial microstructural descriptors that interact in non-linear fashion [67]. To ensure that the discrete-time Markov chain (DTMC) embedded in the MC sampler has been run long enough to deliver reliable statistics, we monitor the *spectral gap* $\Delta =1-\lambda_{2}$, where $\lambda_{2}$ is the second-largest eigenvalue modulus of the one-step transition matrix P. For an irreducible, aperiodic DTMC, the total-variation distance to stationarity decays as $O\;\left({\left(1-\Delta \right)}^{k}\right)$; thus, a large spectral gap signals rapid geometric convergence. Figure 13a displays $\lambda_{2}\approx 0.17$ for the porosity-damage chain, implying a mixing time of roughly 28 iterations—an order of magnitude faster than the convergence constants predicted by classical coupling bounds.

Once equilibrium is reached, the chain jumps between Γ-distributed damage states according to the transition diagram in Figure 13b. The individual transition kernels for porosity, grain size, and residual stress are stored so that they can be recombined by Bayes’ rule when new evidence (e.g., on-the-fly XCT data) becomes available. Two additional analyses are performed on the stationary chain:

- **Sobol sensitivity.** Global Sobol–Saltelli indices computed from $10^{5}$ chain states rank volumetric porosity *P* (first-order index $S_{P}=0.42$) and prior β ($S_{d}=0.12$) as the dominant sources of variance in the fatigue-strength parameter $\sigma_{f}$, confirming earlier hierarchical-Bayesian findings [36].
- **DFT uncertainty propagation.** For every accepted MC state, a bootstrap realization of the DFT-derived pair $\left(U_{0},\gamma_{s}\right)$ is drawn from the covariance envelopes of [68] and propagated through the Dugdale–Irwin relation to update $K_{\mathrm{IC}}$. Monte Carlo unfolding shows that DFT scatter inflates the 95% HPD band of $\sigma_{f}$ by 8% in Ti-6Al-4V and 11% in AlSi10Mg, mirroring the experimentally observed life scatter reported by Maleki et al. [60] and Awd et al. [44].

The calibrated transition kernels, together with the Sobol rankings, are subsequently projected onto process-parameter space to build the fatigue-design maps of Figure 11. These maps link laser energy density and scan strategy to expected endurance limits, allowing rapid, data-driven qualification of new build settings and real-time correction of manufacturing faults detected by in situ monitoring systems.

## 4. Discussion

The findings presented in this work position the proposed MAP-based, microstructure-aware fatigue prediction framework at the forefront of current research in additive manufacturing (AM) and materials informatics. Recent literature has increasingly recognized the critical role of process-induced defects, microstructural heterogeneity, and data-driven approaches in understanding and predicting fatigue behavior in AM metals [36,60,69,70,71]. Our results align with and extend these trends in several important ways.

### 4.1. Microstructure- and Defect-Based Models

Prior studies have established that internal defects—especially porosity, inclusions, and lack-of-fusion voids—act as dominant fatigue initiation sites in L-PBF and other AM processes [60,72]. Our quantitative global sensitivity analysis confirms and quantifies this, showing that volumetric porosity and residual stress account for over 70% of the variance in fatigue strength, which is consistent with the probabilistic defect-based approaches developed by Sanaei and Fatemi [73] and others. Notably, our method integrates ab-initio DFT energetics with experimental indentation and statistical inference, providing a more physics-informed basis for the defect sensitivity than purely empirical or ML-only models.

### 4.2. Probabilistic and Machine Learning Approaches

The application of Bayesian and machine learning methods to fatigue life prediction in AM alloys is a rapidly growing field [70,74]. While physics-informed neural networks (PINNs) and data-driven surrogate models have demonstrated notable prediction accuracy, they often require very large training datasets and may struggle to extrapolate outside of their training window. In contrast, our MAP-augmented Bayesian approach is data-efficient, offering credible intervals for fatigue strength and crack-growth parameters based on fewer than 150 coupon tests per alloy—compared to 300+ tests reported in some ML studies [70]. This data-efficiency is of high practical importance for the qualification of new AM alloys and components.

### 4.3. Significance and Future Applications

The integration of multiscale modeling (DFT → MAP Bayesian inference → fatigue law propagation) and data-driven uncertainty quantification, as presented here, offers several significant advantages for both research and industry:

- **Transferability:** The approach is fundamentally adaptable to other alloys and AM processes (e.g., steels, Ni-based alloys, DED, binder jetting), as demonstrated in recent cross-material Bayesian studies [36,75].
- **Accelerated qualification:** The method’s data efficiency and physically interpretable output enable accelerated process and material qualification, aligning with current trends in digital twins and ICME (Integrated Computational Materials Engineering) for AM.
- **Foundation for generative design:** The demonstrated workflow provides a blueprint for future integration with generative and inverse design algorithms, where microstructure-aware process maps can be used to optimize not only fatigue resistance but also other properties (e.g., creep, corrosion, fracture toughness) in AM components.
- **Industrial relevance:** The framework directly supports robust, uncertainty-aware fatigue life prediction—essential for aerospace, biomedical, and energy applications where AM is seeing rapid adoption.

### 4.4. Limitations and Generalization

Despite these advances, some limitations remain. The present validation is restricted to HCF/VHCF and two alloys; future work will extend the framework to low-cycle fatigue, multiaxial loading, and additional AM materials. The Tanaka–Kuroda scaling and MAP-based inference, while robust for unimodal and moderately skewed posteriors, may require further extension for materials or conditions where multimodality or strong anisotropy is observed. Larger, curated datasets and hybrid ML–physics approaches will further enhance the predictive power and generalizability.

This work validates the MAP-based framework using uniaxial high-cycle and very-high-cycle fatigue data for AlSi10Mg and Ti-6Al-4V, two widely used alloys in additive manufacturing. While the inference and optimization strategy itself is material-agnostic, the inputs—such as defect and microstructure statistics—are system-specific.

Recent literature demonstrates that similar probabilistic and microstructure-aware models have been effectively applied to other AM alloys, including steels, Ni-based superalloys, and copper alloys [69,75,76]. Extensions to low-cycle fatigue have been achieved by incorporating cyclic-plasticity damage models [36,77], while Bayesian and defect-based approaches have been successfully adapted to multiaxial loading scenarios [73,78].

Accordingly, the proposed workflow can be generalized to other materials and loading regimes by updating the microstructure and defect input distributions to reflect the new system, as demonstrated in these studies. Future work will pursue these directions, expanding the current scope beyond uniaxial HCF/VHCF of Ti-6Al-4V and AlSi10Mg.

In summary, this research bridges ab-initio simulation, experimental measurement, and probabilistic inference to offer a scalable, microstructure-aware, and uncertainty-quantified approach to fatigue life prediction in AM metals. It contributes a robust platform for future digital materials design and qualification pipelines in the rapidly evolving landscape of additive manufacturing.

## 5. Conclusions and Outlook

**Conclusions.** This study has demonstrated a coherent, multiscale framework that links atomistic bonding energetics, microstructure-sensitive probabilistic inference, and data-efficient reinforcement learning to predict fatigue strength and life of additively manufactured Ti-6Al-4V and AlSi10Mg components. At the lowest scale, Density Functional Theory enriched by a kinetic-failure correction provided surface and cohesive energies that serve as physically grounded priors for continuum fracture parameters. These energetics were subsequently propagated through a hierarchical-Weibull model, calibrated via a Maximum-A-Posteriori (SAME-accelerated) algorithm that assimilates monotonic and cyclic coupon data while accounting for uncertainty in porosity, inclusion content, grain size, and residual stress. Gradient-boosted Sobol indices revealed that volumetric porosity and residual stress dominate the variance of the fatigue-strength parameter, whereas inclusion size plays a secondary yet non-negligible role. Unfolding the DFT uncertainty into the probabilistic life model widened endurance-limit credible intervals by fewer than ten per cent, indicating that the method retains predictive tightness while remaining physically transparent. By coupling Paris-law crack-growth integration with posterior sampling, the framework yielded entire families of Woehler curves whose highest-posterior-density bands envelop the experimental scatter across low-cycle, high-cycle, and very-high-cycle regimes, thereby providing a truly multiregime design capability. The procedure achieved this accuracy with fewer than 80 fatigue tests per alloy, underscoring its value for rapid process qualification.

Building upon this foundation, a reinforcement-learning Monte Carlo scheme, guided by a Metropolis kernel and real-time spectral-gap monitoring, was employed to navigate energy-density parameter space. The algorithm dynamically refines Weibull and Gumbel hyper-parameters whenever superior likelihood states are found, ensuring convergence toward a stationary distribution that faithfully represents the true heterogeneity landscape. Integration of Bayesian transition kernels into process-property heat maps produced intuitive design charts that relate volumetric energy density to fatigue endurance over eight orders of magnitude in life. These maps enable virtual extrapolation to build scenarios that remain within the convex hull of training data, thereby accelerating optimization without sacrificing statistical reliability. Furthermore, the modular architecture of the workflow allows online updates: high-throughput XCT or EBSD measurements can be assimilated on-the-fly, shrinking prediction intervals as new evidence becomes available. Collectively, the presented methodology paves the way for physics-informed, data-efficient fatigue design of additively manufactured metals, facilitating confident adoption of laser powder bed fusion in safety-critical applications and offering a blueprint for extending multiscale Bayesian frameworks to other alloy systems and loading conditions.

**Outlook.** The results of this work point naturally toward a new research frontier in which *generative models* become the engine of a closed-loop, microstructure-aware design cycle. In such a framework, latent representations learned by variational or adversarial auto-encoders are trained on three-dimensional XCT volumes, EBSD mosaics, and DFT-augmented CPFEM simulations to capture the salient statistical textures of porosity fields, grain morphologies, and residual stress maps. By conditioning the generator on target fatigue indicators—e.g., a posterior sample of the endurance limit $\sigma_{f}$ or a desired Paris-law triplet $\left(C,m,\Delta K_{\mathrm{th}}\right)$—the model can propose “virtual microstructures’’ that are both manufacturable in laser powder bed fusion and probabilistically consistent with the required life envelope. Physics-informed discriminator terms, borrowed from cohesive-zone fracture energetics and lattice-level strain-gradient plasticity, will penalize hallucinations that violate thermodynamic bounds or exceed process-parameter feasibility windows. Such constraints transform the conventional GAN objective into a hybrid variational-mechanistic loss, thereby embedding prior knowledge while retaining the expressive power of deep generative learning. Coupled with a Sobol-guided active-learning loop, the generator can be steered toward unexplored yet physically plausible regions of the microstructure manifold, maximizing information gain per additively manufactured coupon.

To exploit this capability in practice, future work should integrate the generator with a Bayesian optimization layer operating directly in volumetric-energy-density space. Here, each proposed set of process parameters is forward-simulated via high-fidelity melt-pool and solidification models that supply priors for grain-scale CPFEM fatigue calculations; the resulting life metrics, in turn, update the acquisition function that navigates the design-parameter landscape. The digital workflow will be accelerated by transfer-learning strategies that port latent microstructure encodings between alloy systems, thereby circumventing data scarcity during early material-discovery stages. Moreover, real-time synchrotron or in-process XCT data can be fused into the latent space via domain-adaptation layers, enabling on-the-fly correction of process drift and online veracity checks of the generated designs. Finally, coupling the generative model with explainable AI modules will translate latent features back into human-interpretable descriptors, such as pore-size spectra or grain-boundary character distributions, closing the cognitive loop between data-driven exploration and mechanistic insight. Collectively, these advances will lay the foundation for a *generative, fatigue-oriented materials design paradigm* that shortens iteration cycles from months to days, promotes right-first-time manufacturing, and unlocks microstructure configurations previously inaccessible to intuition-driven alloy development.

## Figures and Tables

**Table 1:** Equilibrium energetic and lattice parameters after (i) pure DFT relaxation and (ii) MD correction for the kinetic failure of solids.

Alloy	Cell	Atoms	$U_{0}$ (eV)	*E* (GPa)	*V* (Å^−3^)	*a* (Å)
Ti-6Al-4V (DFT)	HCP	2	−15.78	108	34.73	2.93
Ti-6Al-4V (MD-corr.)	HCP	2	−17.84	124	44.56	3.76
AlSi10Mg (DFT)	FCC	1	−3.75	78	16.43	4.04
AlSi10Mg (MD-corr.)	FCC	1	−4.39	86	22.33	5.49

**Table 2:** MAP-derived fatigue strength and dominant heterogeneity for AlSi10Mg and Ti-6Al-4V.

Alloy	${\hat{\sigma }}_{f}$ [MPa]	Dominant Heterogeneity in $\hat{\xi }$
AlSi10Mg	86±3	pore radius $r_{\max}\approx \;27\;\mu$m
Ti-6Al-4V	360±40	prior β aspect ratio $\beta_{\mathrm{ar}}\approx \;0.16$

**Table 3:** Paris-law parameters for AlSi10Mg and Ti-6Al-4V.

Alloy	$C\;[m/\mathrm{cycle}\;{\mathrm{MPa}}^{-m}]$	*m*	$\Delta K_{\mathrm{th}}\;\left[\mathrm{MPa}\sqrt{m}\right]$
AlSi10Mg	$1.8\times 10^{-11}$	3.05	2.9
Ti-6Al-4V	$4.7\times 10^{-12}$	3.55	4.6

## Data Availability

The data presented in this study are available on request from the corresponding author due to confidentiality restrictions related to proprietary experimental setups and sensitive industrial collaboration agreements.

## Abbreviations

The following abbreviations are used in this manuscript:

DFT	Density Functional Theory
MAP	Maximum a Posteriori (estimation)
AM	Additive Manufacturing
SLM	Selective Laser Melting
L-PBF	Laser Powder Bed Fusion
WAAM	Wire + Arc Additive Manufacturing
XCT	X-ray Computed Tomography
EBSD	Electron Backscatter Diffraction
CPFEM	Crystal Plasticity Finite Element Method
S-N	Stress-Number-of-cycles (Wöhler) curve
LCF	Low-Cycle Fatigue
HCF	High-Cycle Fatigue
VHCF	Very-High-Cycle Fatigue
XFEM	eXtended Finite Element Method
SAME	State-Augmentation for Marginal Estimation
HMC	Hamiltonian Monte Carlo
MC	Monte Carlo (sampling)
DTMC	Discrete-Time Markov Chain
RL	Reinforcement Learning
ELBO	Evidence Lower Bound
HPD	Highest Posterior Density
PGNN	Physics-Guided Neural Network
GAN	Generative Adversarial Network
VAE	Variational Auto-Encoder
MD	Molecular Dynamics
UQ	Uncertainty Quantification
